# Combined QTL mapping and RNA-Seq profiling reveals candidate genes associated with cadmium tolerance in barley

**DOI:** 10.1371/journal.pone.0230820

**Published:** 2020-04-16

**Authors:** Behnam Derakhshani, Hossein Jafary, Bahram Maleki Zanjani, Karim Hasanpur, Kohei Mishina, Tsuyoshi Tanaka, Yoshihiro Kawahara, Youko Oono

**Affiliations:** 1 Department of Agronomy & Plant Breeding, Faculty of Agriculture, University of Zanjan, Zanjan, Iran; 2 Breeding Material Development Unit, Institute of Crop Science, National Agriculture and Food Research Organization (NARO), Tsukuba, Ibaraki, Japan; 3 Iranian Research Institute of Plant Protection, Agricultural Research, Education and Extension Organization (AREEO), Tehran, Iran; 4 Department of Animal Science, Faculty of Agriculture, University of Tabriz, Tabriz, Iran; 5 Plant Genome Research Unit, Institute of Crop Science, NARO, Tsukuba, Ibaraki, Japan; 6 Breeding Informatics Research Unit, Institute of Crop Science, NARO, Tsukuba, Ibaraki, Japan; 7 Bioinformatics Team, Advanced Analysis Center, NARO, Tsukuba, Ibaraki, Japan; North Dakota State University, UNITED STATES

## Abstract

The high toxicity of cadmium (Cd) and its ready uptake by plants has become a major agricultural problem. To investigate the genetic architecture and genetic regulation of Cd tolerance in barley, we conducted quantitative trait loci (QTL) analysis in the phenotypically polymorphic Oregon Wolfe Barley (OWB) mapping population, derived from a cross between Rec and Dom parental genotypes. Through evaluating the Cd tolerance of 87 available doubled haploid lines of the OWB mapping population at the seedling stage, one minor and one major QTL were detected on chromosomes 2H and 6H, respectively. For chlorosis and necrosis traits, the major QTL explained 47.24% and 38.59% of the phenotypic variance, respectively. RNA-Seq analysis of the parental seedlings under Cd treatment revealed 542 differentially expressed genes between Cd-tolerant Rec and Cd-susceptible Dom genotypes. By analyzing sequence variations in transcribed sequences of the parental genotypes, 155,654 SNPs and 1,525 InDels were identified between the two contrasting genotypes and may contribute to Cd tolerance. Finally, by integrating the data from the identified QTLs and RNA-Seq analysis, 16 Cd tolerance-related candidate genes were detected, nine of which were metal ion transporters. These results provide promising candidate genes for further gene cloning and improving Cd tolerance in barley.

## Introduction

Cadmium (Cd) contamination of agricultural soils has been receiving worldwide attention due to its entry into food crops such as barley (*Hordeum vulgare*), wheat (*Triticum aestivum*) and rice (*Oryza sativa*) [1, 2]. In plants, Cd is reported to reduce chlorophyll content, alter nitrogen metabolism and inhibit the photosynthetic apparatus [3, 4]. The most apparent symptom of Cd toxicity in plants is leaf chlorosis, and increasing the duration of treatment and/or the Cd concentration leads to the transition from leaf chlorosis into necrosis [5, 6]. Plants have evolved diverse mechanisms to adapt to Cd-contaminated environments, such as extrusion across plasma membrane, chelation in the cytosol and vacuolar sequestration [7]. Transporters with heavy metal binding domains such as pleiotropic drug resistance-type ATP-binding (ABC) protein (PDR9) and multi-antimicrobial extrusion protein (MatE), are involved in efflux pumping of Cd at the plasma membrane [8, 9]. Heavy metal transporting ATPase 3 (HMA3), which is located in the tonoplast, contributes in sequestrating Cd into vacuoles [10]. It is also reported that Zn transporters such as Zrt (zinc-regulated transporter)/IRT-like protein 1 (ZIP) and the cation efflux family (Cation_efflux) proteins are capable of transporting Cd ions [11, 12]. However, the molecular mechanisms underlying Cd tolerance and accumulation in barley are poorly understood.

Quantitative trait loci (QTL) mapping is a commonly used method to identify candidate genes associated with phenotypic traits. The Oregon Wolfe Barley (OWB) doubled haploid (DH) mapping population, one of the most widely used mapping populations, is a set of 94 spring barley DH lines derived from the F_1_ of a cross between the parental genotypes Rec and Dom using the *Hordeum bulbosum* method [13]. Navakode et al. [14] reported minor QTLs for aluminum (Al) tolerance located on chromosomes 2H, 3H and 4H in the OWB mapping population under different Al concentrations. Another study on the OWB mapping population identified a major QTL for salt tolerance on chromosome 5H, with a peak marker *GBS0318* located at 50.44 centimorgans (cM) [15]. The parents of the OWB mapping population have been developed by systematically crossing recessive alleles into one parent (Rec) and dominant alleles into the other parent (Dom) to produce dominant and recessive morphological marker stocks [16]. It has been reported that the parental genotypes Rec and Dom showed different responses in terms of germination under salinity stresses [17].

Recently, RNA-Seq using deep-sequencing technologies has been the technology of choice for analysis of a whole transcriptome, detecting low abundance transcripts and genetic variants such as single nucleotide polymorphisms (SNPs) and insertions/deletions (InDels) [18, 19]. The main objectives of our study are to: (1) dissect the genetic mechanism of Cd tolerance by QTL mapping using the OWB mapping population; (2) dissect the molecular mechanism of Cd tolerance by characterizing the expression profile and sequence polymorphism in the parental seedlings under Cd stress using RNA sequencing technology; and (3) integrate the QTL mapping and RNA sequencing to identify potential candidate genes for further functional and mechanism studies.

## Materials and methods

### Plant materials and linkage map construction

The OWB is a DH mapping population developed in North America and is a reference mapping population subjected to extensive genotyping and phenotyping. The seeds of the OWB population were kindly provided by Prof. Patrick Hayes of Oregon State University who is responsible for the population. The genotype data of the OWBs including 3,714 markers were downloaded from the Oregon State University (OSU) Barley Project web site (https://barleyworld.org). The order of the markers was determined using JoinMap v.5 software (https://kyazma.nl/index.php/JoinMap/), and the recombinant markers in each chromosome were selected, which are shown in S1 Table.

### QTL mapping and Cd treatment

To collect quantitative data for QTL analysis, both parents (Rec and Dom) and 87 available lines of the OWB mapping population were grown in ¼ strength Hoagland nutrient solution in greenhouse conditions at the Agricultural and Natural Resources Research and Education Center, Zanjan, Iran. Each sample had three biological replicates. About 14 days after the lines were planted, 5 mM Cd (CdCl_2_.H_2_O) treatment was started, and 10 days later, the percentage of the leaves showing symptoms like chlorosis and necrosis were estimated for each line in three replicates. Plant leaves were thoroughly washed with tap and deionized water and were oven-dried (72 °C) to a constant weight. The dried leaves were weighed and ground into powder using an acid digestion method, followed by the measurement of Cd concentration with an atomic absorption spectrophotometer (GBC Avanta, Australia).

QTL Cartographer v2.5 was used to map QTL by composite interval mapping (CIM) analysis with a 1,000-permutation test to estimate the significance threshold of the logarithm of odds (LOD) score [20]. Pearson’s correlation coefficient analysis was performed and visualized by the ggpairs function in the R/GGally package (https://cran.r-project.org/web/packages/GGally/index.html).

### Cd treatment of parents for RNA-Seq analysis

Cd-tolerant Rec and Cd-susceptible Dom seeds were germinated and grown by hydroponic culture in ¼ strength Hoagland nutrient solution under a 16/8 h day/night photoperiod and 27 °C /18 °C day/night temperatures [21]. After 14 days, seedlings of uniform size were treated with Hoagland nutrient solution with or without 5 mM Cd for 2 h. Seedlings grown in Cd-free solution were treated as controls (CKs). Following Cd treatment, shoot samples were collected and immediately frozen in liquid nitrogen and stored at −80 °C.

### RNA isolation and RNA sequencing

Total RNA was isolated from shoot samples of the parental genotypes Rec and Dom under Cd and CK treatments after 2 h using the RNeasy Plant Mini Kit (Qiagen, Hilden, Germany) according to the manufacturer’s protocol. For RNA-Seq analysis, we used two biological replicates for each treatment (Cd and CK) in each genotype (Rec and Dom). High-quality total RNA samples were sent to the BGI-Hong Kong NGS Lab and sequenced with an Illumina HiSeq sequencing platform.

### Reads preprocessing and mapping

Estimation of RNA-Seq data quality was performed by FastQC v0.10.1 (http://bioinformatics.bbsrc.ac.uk/projects/fastqc). Using Trimmomatic v0.32, low-quality bases at both the 5′ and 3′ ends of each read were trimmed with the settings minimum read length > 70, sliding window = 25 bp and average quality > 25 [22]. The barley genome assembly (Hordeum_vulgare. Hv_IBSC_PGSB_v2.dna.toplevel.fa) was downloaded from the Ensembl Plants database (https://plants.ensembl.org/Hordeum_vulgare/Info/Index) and a genome index was constructed with the Bowtie v2.3.4.1 software [23]. The clean reads were mapped to the barley genome assembly using the Tophat v2.1.1 and the alignments were sorted using SAMTools v1.8 [24, 25]. The obtained RNA-Seq data were submitted to the Sequence Read Archive of the National Center for Biotechnology Information (NCBI) with the accession number SRP165638.

### Analysis of differentially expressed genes (DEGs) and Kyoto Encyclopedia of Genes and Genomes (KEGG) pathway functional annotation

Read counts for each gene were generated using HTSeq-counts v0.9.1 [26]. Identification of DEGs was performed with the R package DESeq2 v3.7, and DEGs with an adjusted *p*-value (padj) ≤ 0.05 and expression level of |log_2_ fold change| ≥ 1 were selected for further analyses [27]. To get functional annotations for the genes, a BLASTP search was performed against the NCBI Nr protein database and Pfam with an E-value 1.0e-8 cut-off. After choosing the best hits, KEGG pathway enrichment analysis was performed for the up- and downregulated DEGs separately with KOBAS software (adjusted *p*-value ≤ 0.05) [28].

### Variant calling

To study the association between polymorphic sites and Cd tolerance, variants were detected in each genotype by comparing the sequenced reads to the barley genome reference. After mapping the obtained clean reads to the barley genome assembly using the Tophat v2.1.1, all four BAM files for each genotype were merged into one file. Variant calling was performed using the *mpileup* function in SAMTools v1.8 and the BCFTools package was used to filter the variants with a minimum Phred quality of 20 and a maximum read depth of 100 [25]. Heterozygous variants were also filtered out and the effects of the candidate variants on amino acids were predicted with SnpEff [29].

### qRT-PCR verification of RNA-Seq data

To confirm the expression of the Cd-responsive genes in shoot samples, qRT-PCR analysis was performed with three technical replicates. RNA samples were reverse transcribed into cDNA using a PrimeScript RT reagent Kit (Takara, Japan) according to the manufacturer’s instructions. The THUNDERBIRD SYBR qPCR Mix kit (TOYOBO, Japan) was used for qRT-PCR quantification on a Bio-Rad CFX96 Real Time PCR system (Bio-Rad, Hercules, CA, United States). The barley *Actin* gene was used as an internal control and the relative gene expression was calculated using the 2^−ΔΔCt^ method. The information about designed primer pairs is shown in S2 Table.

## Results

### Morphological symptoms of Cd stress in the parental genotypes

The Cd-tolerant Rec and Cd-susceptible Dom genotypes are parents of the OWB DH mapping population [13], and are shown in S1A Fig. Phenotypic characters of 14-day-old parental genotypes, which were treated for nine days under 5 mM Cd concentration are shown in S1B Fig. The usage of high Cd concentration (4 mM CdCl_2_) has been reported in a transcriptome study of cotton (*Gossypium hirsutum*) [30]. Shoots of Dom genotype showed chlorosis and necrotic spots, which progressed to drying of the leaves and stunting of the seedlings. It is reported that in response to Cd stress, reactive oxygen species (ROS) can be formed and lead to necrotic cell death in plants [31]. The severity of the chlorosis and necrosis symptoms on Rec shoots were less intense in comparison with Dom shoots. The observed symptoms under Cd exposure in our study were consistent with a previous report in rice [32]. Due to the contrasting phenotype of Rec and Dom under Cd stress, these two parents of the OWB mapping population were chosen for further RNA-Seq analysis, as a highly Cd-tolerant and susceptible genotypes.

### Phenotypic variation and correlation analysis between the leaf chlorosis and necrosis with Cd concentration

**Fig 1:**
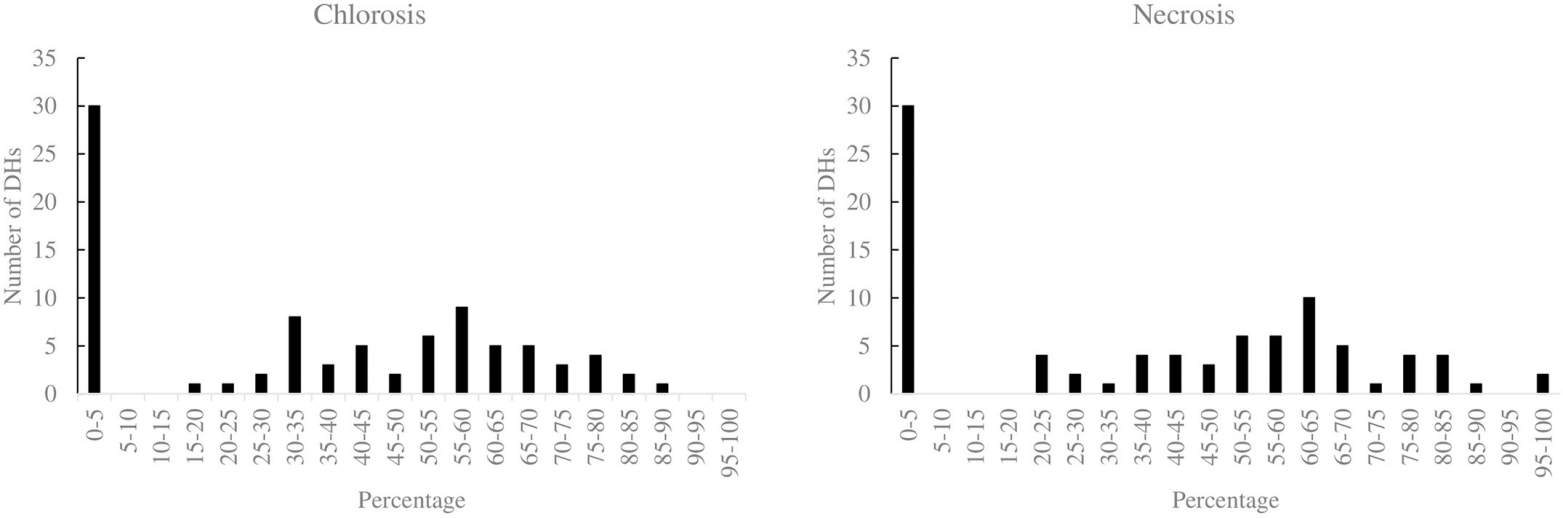
Phenotypic distribution of the chlorosis and necrosis traits in the OWB population grown under Cd treatment. The unit of x-axis represents the percentage of leaves in each line showing stress symptoms. The unit of y-axis represents the number of DH lines. The data are represented in three replicates.

In the OWB mapping population, bi-modal distribution patterns for the chlorosis and necrosis traits in different replicates were observed (Fig 1), which indicated the segregation of a QTL with a large effect. Pearson correlation coefficient was determined to evaluate the relatedness of the chlorosis and necrosis traits with the leaf Cd concentration in the OWB mapping population (S2 Fig). A strong positive correlation (r = 0.97) was detected between the chlorosis and necrosis traits in the OWB mapping population. In contrast, very weak correlations between the abovementioned traits with the leaf Cd concentration (r = 0.26 and r = 0.28, respectively) were observed in the OWB lines. Therefore, we assumed that tolerant (without the chlorosis and necrosis symptoms) and susceptible lines (with the chlorosis and necrosis symptoms) in the OWB mapping population did not differ in root-based exclusion or translocation of Cd from the roots to the shoots, but probably tolerant lines developed shoot-based mechanisms such as extrusion or vacuolar sequestration [33].

### QTL analysis for Cd tolerance at the seedling stage

**Fig 2:**
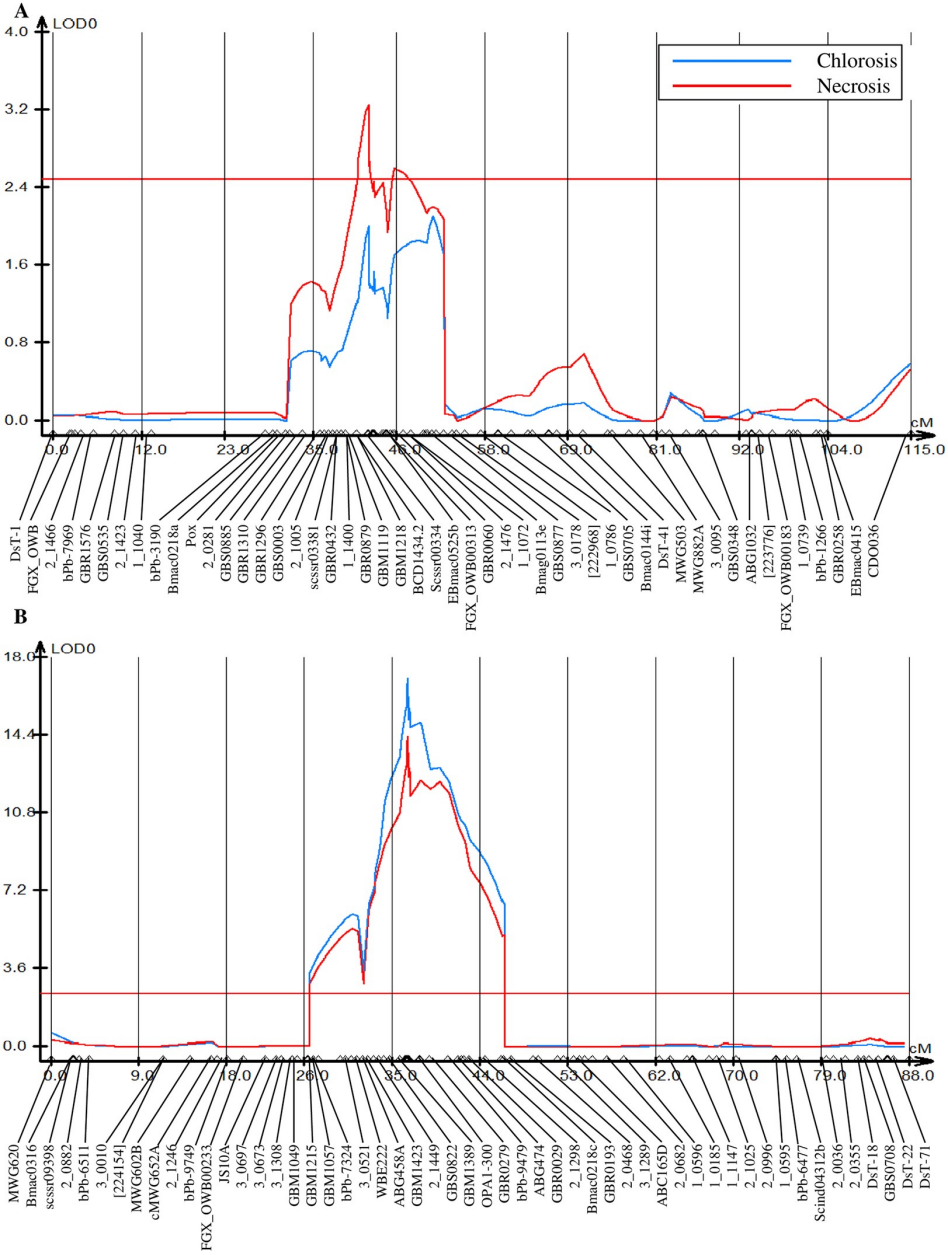
QTL scanning curves for the chlorosis and necrosis in the OWB population. QTL curves on chromosomes (A) 2H and (B) 6H of the OWB population. The horizontal axis represents the position of markers. The vertical axis represents the LOD score. R: Replicate.

Based on the genotypic analysis of 87 DH lines using 3,714 markers (https://barleyworld.org), one minor and one major QTL associated with Cd stress response were identified (Fig 2A and 2B and S3 Fig). The minor QTL located on chromosome 2H was only detected by necrosis index (Fig 2A). However, the major QTL located on chromosome 6H was detected using the chlorosis and necrosis as indexes (Fig 2B). Of these, the minor QTL explained 6.39% of the phenotypic variation of the necrosis trait (Table 1). The major QTL explained 47.24% and 38.59% of the phenotypic variation of the chlorosis and necrosis traits, respectively. Moreover, based on the gene annotation information of the barley reference genome (https://plants.ensembl.org/Hordeum_vulgare/Info/Index), chromosomal regions of the minor and major QTL harbored a total of 1,881 and 2,760 genes located between the *scssr00334-1_1072* and *GBM1215*-*GBR0193* markers, respectively (S3 Table).

**Table 1:** QTL peaks on 2H and 6H for Cd-tolerance in the OWB mapping population.

Trait	Chromosome	Position (cM)	Genomic region (Mb)	LOD	Flanking markers	R^2^ (%) ^a^	Additive effect
Chlorosis	6H	26.38–46.19	24.64–490.06	17.03	*GBM1215-GBR0193*	47.24	20.40
Necrosis	2H	44.74–47.94	533.46–709.52	3.25	*scssr00334-1_1072*	6.39	8.30
	6H	26.38–46.19	24.64–490.06	14.37	*GBM1215-GBR0193*	38.59	20.44

^a^R^2^: Percentage of phenotypic variation explained by the QTL

### RNA-Seq analysis and mapping

**Fig 3:**
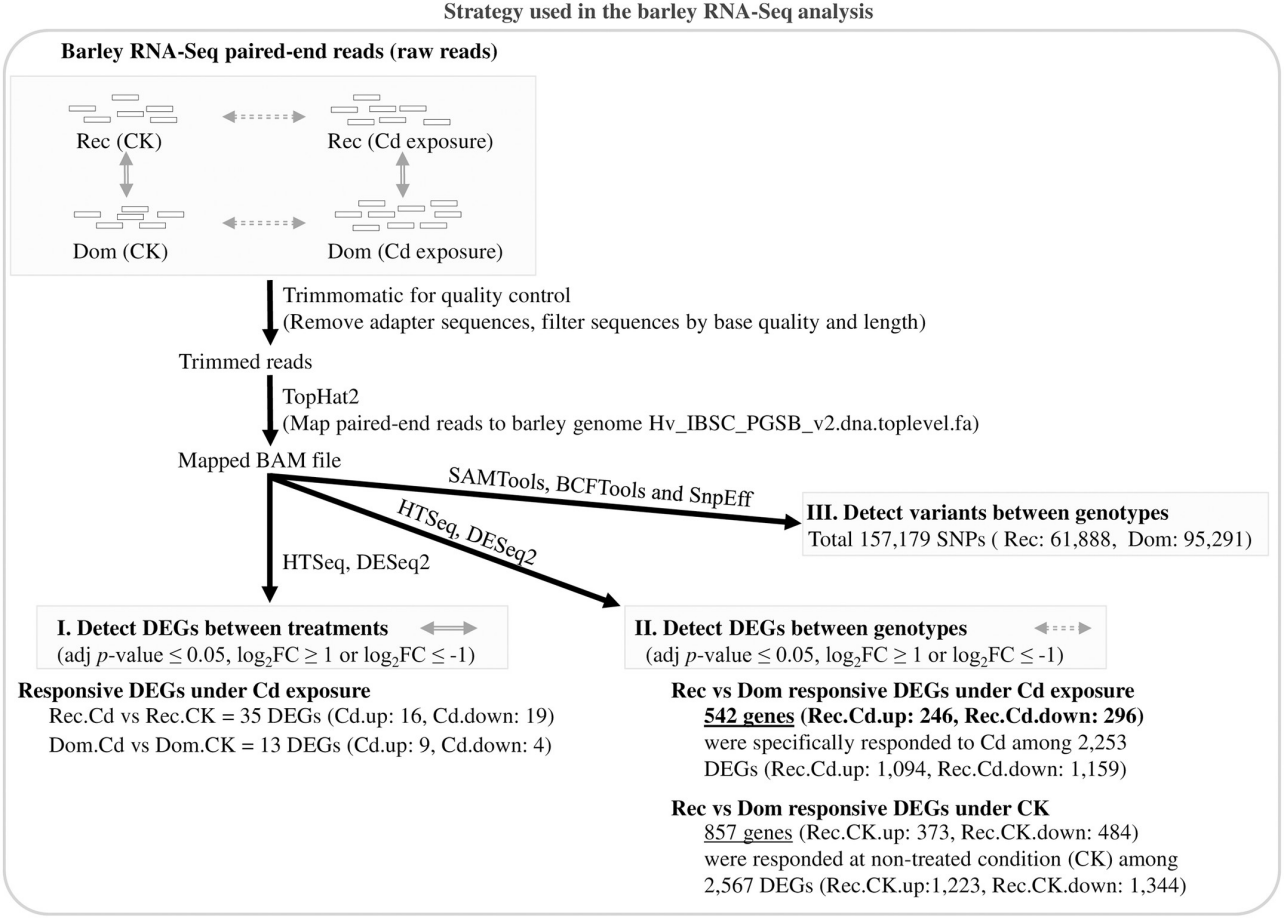
General workflow used in the barley RNA-Seq analysis. After quality control of raw reads by FastQC, we used Trimmomatic to trim low-quality bases. The Bowtie v2.3.4.1 software was used to make a barley genome index. The clean reads obtained from the RNA-Seq data were mapped to the barley genome assembly using Tophat v2.1.1 and the alignments were sorted by SAMTools v1.8. HTSeq-counts v0.9.1 was used to generate read counts of each gene, and DESeq2 v3.7 was used to identify DEGs between treatments and genotypes. Detection of variants was performed using SAMTools v1.8, BCFTools and snpEff pipeline.

To better understand the barley transcriptomic response to Cd stress, we performed RNA-Seq analysis of 14-day-old parental seedlings that were treated for 2 h with Cd or without Cd (CK). After quality control and trimming of low-quality reads, an average of 34.18 and 33.38 million clean reads was obtained and among them about 31.74 (91.58%) and 30.76 (90.83%) million reads were mapped to the reference genome in Rec and Dom, respectively (Fig 3 and S4 Table). As the ratio of reads mapped to the genome did not differ between Rec and Dom, we considered the reads to be suitable for subsequent analysis.

### Identification and functional annotation of DEGs in pairwise comparisons

**Fig 4:**
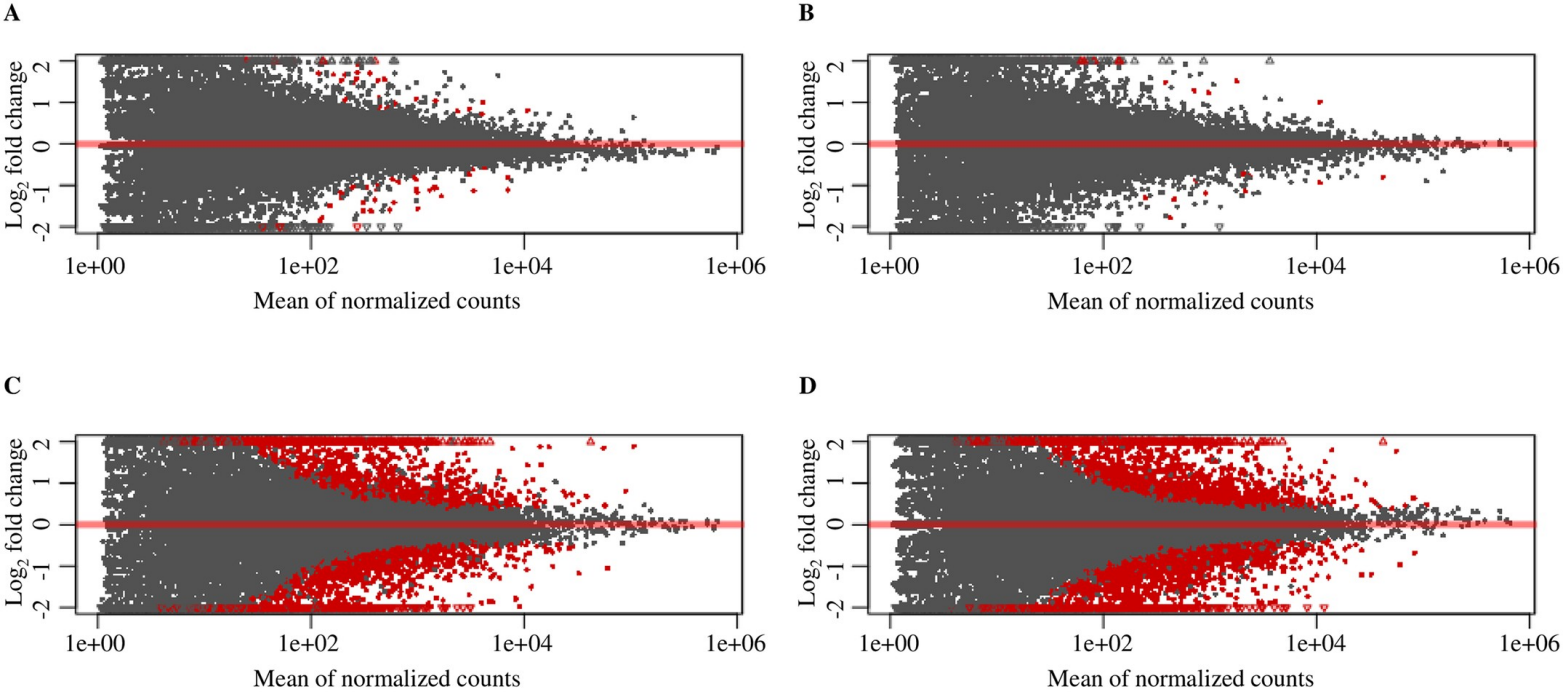
MA plots produced using DESeq2 for differential expression analysis in four comparisons. The x-axis shows the mean normalized read counts and the y-axis shows log_2_ fold changes. Points in red show significant DEGs (Padj ≤ 0.05). The positive area shows upregulated genes and the negative area shows downregulated genes. (A) In Rec genotype, between Cd stress and CK treatment (Rec.Cd vs Rec.CK), (B) in Dom genotype, between Cd stress and CK treatment (Dom.Cd vs Dom.CK), (C) between the two genotypes and under Cd treatment (Rec.Cd vs Dom.Cd), and (D) between the two genotypes and under CK treatment (Rec.CK vs Dom.CK). CK: Control.

MA plot analysis was used to visualize individual gene responses plotted as log_2_ fold change versus mean of normalized counts for the DEGs between treatments (Fig 4A and 4B) and between the two genotypes (Fig 4C and 4D). Volcano plot analysis was used to demonstrate the fold changes in the expression at the x-axis and significance value at the y-axis for the DEGs in the abovementioned comparisons (S4A–S4D Fig). A larger number of significant DEGs were found between genotypes (2,253 and 2,567 DEGs between the two genotypes under Cd and CK treatments, respectively) than between treatments (35 and 13 DEGs in Rec and Dom between Cd and CK treatments, respectively), suggesting that the effect of genotype was bigger than that of treatment. The distribution of DEGs in the four comparisons is shown in Fig 3 and S4E Fig. To widely identify DEGs that may contribute to Cd tolerance in Rec, we examined two categories of Cd-responsive DEGs, including between Cd and CK treatments and between the two genotypes.

In Rec genotype, between Cd stress and CK condition (Rec.Cd vs Rec.CK), 16 and 19 DEGs were up- and downregulated, respectively (Fig 3 and S5 Table). In Dom genotype, between Cd stress and CK condition (Dom.Cd vs Dom.CK), nine and four DEGs were up- and downregulated, respectively (S6 Table). Between the two genotypes and under Cd treatment (Rec.Cd vs Dom.Cd), a total of 1,094 and 1,159 DEGs were up- and downregulated, respectively (S7 Table). A total of 542 DEGs (246 up- and 296 downregulated) were specifically regulated by Cd stress in Rec.Cd vs Dom.Cd and may function in Cd response (S7 Table). Additionally, 857 DEGs (373 up- and 484 downregulated) were only differentially regulated between the two genotypes and under CK treatment in Rec.CK vs Dom.CK, which could be attributed to the genotypic differences (S8 Table).

Both the MA plot and volcano plot analyses suggested that the effect of genotype was bigger than that of treatment (Fig 4A–4D and S4A–S4D Fig). Therefore, to validate the results of RNA-Seq analysis, qRT-PCR was conducted on six randomly selected DEGs with three up- and three downregulated DEGs between the two genotypes under Cd and/or CK treatments (S5A Fig). The expression patterns for six DEGs were generally consistent between RNA-Seq and qRT-PCR data, indicating that RNA-Seq data was reliable (S5B Fig). However, the differences observed in fold changes measured by RNA-Seq and qRT-PCR were consistent with the previous studies [34].

To functionally annotate the genes that were differentially regulated between the treatments and between the two genotypes, we used KEGG pathway enrichment analysis. We could not identify any significantly enriched pathway for the DEGs in Rec.Cd vs Rec.CK comparison. Among specifically upregulated DEGs in Rec.Cd vs Dom.Cd comparison, the top three highly significant pathways (based on adjusted *p*-value) were involved in biosynthesis of secondary metabolites (28 genes), metabolic pathways (37 genes) and alpha linolenic acid metabolism (5 genes) (S9 Table). The top three highly enriched pathways in downregulated DEGs were related to metabolic pathways (36 genes), biosynthesis of secondary metabolites (20 genes) and limonene and pinene degradation (2 genes). The abovementioned KEGG pathways among upregulated DEGs in Rec.Cd vs Dom.Cd have been well-documented to play roles in stress response [35–37], therefore we focused on those DEGs for further analysis and identification of Cd stress responsive genes.

### Detection of variants between the parental genotypes

We investigated the polymorphic sites between the two genotypes by comparing their transcript sequences with the barley reference genome assembly and identified possible variants associated with their different responses to Cd stress. After additional filtration of low-ranked (mostly without impact on protein function) and modifier variants (usually non-coding), 155,654 SNPs (moderate and high impact) and 1,525 InDels (high impact) were detected between the two genotypes by mapping to the Morex genome. The most frequent term was missense variant, which included 59,245 SNPs in Rec and 91,474 in Dom (Fig 3, Table 2 and S10 Table). These results revealed a big difference in the parental genome structures due to the occurrence of multiple variants, which may contribute to protein functions and the drastic parental morphological/physiological differences.

**Table 2:** Summary of variant calling between Rec and Dom.

Variant category	Effect (sequence ontology)	Impact	Rec	Dom	All
SNPs	Missense_variant	MODERATE	59,245	91,474	150,719
	Missense_variant&splice_region_variant	MODERATE	857	1,421	1,869
	Stop_gained	HIGH	551	641	904
	Splice_acceptor_variant&intron_variant	HIGH	263	342	893
	Splice_donor_variant&intron_variant	HIGH	305	363	668
	Stop_lost	HIGH	117	221	338
	Start_lost	HIGH	57	140	155
	Stop_lost&splice_region_variant	HIGH	7	13	70
	Stop_gained&splice_region_variant	HIGH	14	20	34
	Start_lost&splice_region_variant	HIGH	2	1	3
	5_prime_UTR_truncation&exon_loss_variant	MODERATE	0	1	1
	Total SNPs	-	61,418	94,637	155,654
InDels	Frameshift_variant	HIGH	448	627	1,484
	Frameshift_variant&splice_region_variant	HIGH	6	13	19
	Frameshift_variant&stop_lost	HIGH	15	4	11
	Frameshift_variant&stop_gained	HIGH	0	8	8
	Frameshift_variant&start_lost	HIGH	0	2	2
	Frameshift_variant&stop_lost&splice_region_variant	HIGH	1	0	1
	Total InDels	-	470	654	1,525
	Total variant sites		61,888	95,291	157,179

### Integration of RNA-Seq data with QTLs

**Fig 5:**
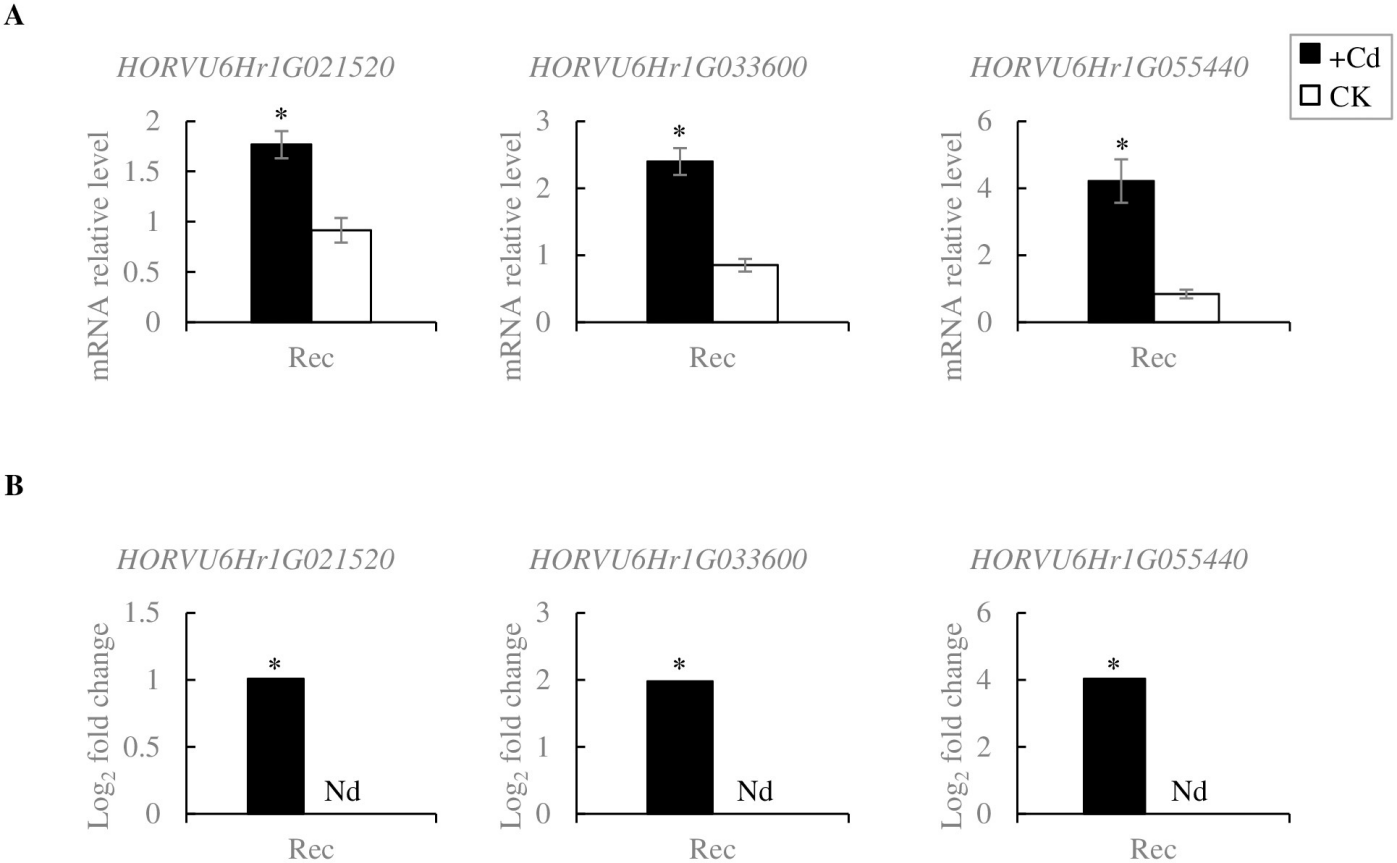
Validation of expression profile for three candidate genes by qRT-PCR. The expression profile of the three candidate genes detected by (A) qRT-PCR and (B) RNA-Seq techniques, which were co-located with the major QTL on 6H chromosome. The relative expression levels of the selected genes were compared with Dom genotype and normalized using an internal control (*Actin*) and calculated based on the 2^−ΔΔCt^ method. For the qRT-PCR data, the mean ± standard error of three technical replicates are represented. Asterisks indicate levels of significance of differential expression tested by the Student’s t-test (* p ≤ 0.05, ** p ≤ 0.01). Nd: No data.

To further understand the roles of the detected DEGs and variants associated with Cd stress tolerance and to narrow down the candidate genes, they were integrated with 1,881 and 2,760 genes in the QTLs on chromosomes 2H and 6H identified in the OWB mapping population (S3 Table). A total of 19 DEGs (specifically upregulated in Rec.Cd vs Dom.Cd comparison) and 951 genes harboring variants (seven overlapping genes with DEGs) were co-localized with the detected QTLs (S11 Table). A BLASTP search for sequences with homology to the *Arabidopsis thaliana* led to the identification of 16 Cd stress-related genes on chromosomes 2H and 6H based on three criteria, including upregulated DEGs in Rec.Cd vs Dom.Cd, genes containing variants and the function of genes (metal ion transporters) located in the QTL regions. We identified three oxidative stress-related DEGs located in the major QTL on chromosome 6H, including *peroxidase P7* (*HORVU6Hr1G021520*), *lipoxygenase 2*.*3* (*HORVU6Hr1G033600*) and *glycine rich RNA-binding protein Grp2A* (*HORVU6Hr1G055440*) genes among the 246 Cd-upregulated DEGs in Rec. The upregulation of these candidate genes was confirmed by qRT-PCR and showed similar results to those of RNA-Seq analysis (Fig 5A and 5B).

We selected genes containing variants in the QTL regions, which may cause amino acid substitution, including *zinc transporter 2* (*HORVU6Hr1G018070*), *ABC transporter G family member 36* (*HORVU6Hr1G019080*), *copper-transporting ATPase HMA5* (*HORVU6Hr1G033380*), *peroxidase 39* (*HORVU6Hr1G026230*), *phospholipid hydroperoxide glutathione peroxidase* (*HORVU6Hr1G063830*), *serine/threonine-protein kinase OXI1* (*HORVU6Hr1G054770*) and *mitogen-activated protein kinase kinase kinase YODA* (*HORVU6Hr1G064150*), in the major QTL on 6H chromosome (Table 3 and S11 Table).

**Table 3:** Candidate genes on detected QTLs related to Cd tolerance.

	Gene ID	Locus	Description (nr database)	Pfam description	log_2_FC	Effect (sequence ontology)	No. of transcripts	*A*. *thaliana*	Reference
					Rec.Cd vs Dom.Cd		with variants	Gene ID	
**DEGs**	*HORVU6Hr1G021520*	chr6H:65256521–65259501	Peroxidase P7	Peroxidase	1.01	-	-	*AT5G05340*	-
	*HORVU6Hr1G033600*	chr6H:154877863–154882095	Lipoxygenase 2.3	PLAT; Lipoxygenase	1.98	Missense_variant	9	*AT3G45140*	Smeets et al., 2009
	*HORVU6Hr1G055440*	chr6H:352480530–352481695	Glycine-rich RNA-binding protein GRP2A	RRM_1	4.03	-	-	*AT4G39260*	Schmidt et al., 2010
**Variants**	*HORVU6Hr1G018070*	chr6H:44280074–44296135	Zinc transporter 2	Zip	-	Missense_variant	9	*AT1G55910*	-
	*HORVU6Hr1G019080*	chr6H:50166476–50175300	ABC transporter G family member 36	ABC2_membrane; PDR_assoc; ABC_tran	-	Frameshift_variant, Frameshift_variant&splice_region_variant, Missense_variant	3, 3, 56	*AT1G15520*	Lee et al., 2005
	*HORVU6Hr1G026230*	chr6H:98093152–98095636	Peroxidase 39	Peroxidase	-	Missense_variant	6	*AT4G11290*	-
	*HORVU6Hr1G033380*	chr6H:151875761–151881885	Copper-transporting ATPase HMA5	HMA; E1-E2_ATPase; Hydrolase	-	Missense_variant	1	*AT1G63440*	Andrés-Colás et al., 2006
	*HORVU6Hr1G054770*	chr6H:347811419–347812933	Serine/threonine-protein kinase OXI1	Pkinase	-	Missense_variant	3	*AT3G25250*	Rentel et al., 2004
	*HORVU6Hr1G063830*	chr6H:432105159–432108006	Probable phospholipid hydroperoxide glutathione peroxidase	GSHPx	-	Missense_variant	6	*AT4G11600*	Milla et al., 2003
	*HORVU6Hr1G064150*	chr6H:433701354–433714373	Mitogen-activated protein kinase kinase kinase YODA	Pkinase	-	Missense_variant	11	*AT1G63700*	Moustafa et al., 2008
**Function**	*HORVU2Hr1G082080*	chr2H:596305353–596308274	Metal tolerance protein 1	Cation_efflux	-	-	-	*AT2G46800*	-
	*HORVU2Hr1G082090*	chr2H:596305657–596344620	Metal tolerance protein 1	Cation_efflux	-	-	-	*AT2G46800*	-
	*HORVU6Hr1G033420*	chr6H:152357561–152364886	Zinc transporter ZTP29	Zip	-	-	-	*AT3G20870*	Wang et al., 2010
	*HORVU6Hr1G036800*	chr6H:182926207–182933284	ABC transporter G family member 39	ABC_trans_N; ABC_tran; ABC2_membrane; PDR_assoc	-	-	-	*AT1G15520*	Lee et al., 2005
	*HORVU6Hr1G065390*	chr6H:449421770–449423617	Detoxification 49	MatE	-	-	-	*AT5G19700*	-
	*HORVU6Hr1G066220*	chr6H:458973819–458974889	Heavy metal-associated isoprenylated plant protein 45	HMA	-	-	-	*AT3G56891*	-

In the function category, we identified six proteins with metal ion binding domains, including PF01545 (Cation_efflux, e.g., *HORVU2Hr1G082080*), PF02535 (Zip, *HORVU6Hr1G033420*), PF08370 (PDR_assoc, *HORVU6Hr1G036800*), PF01554 (MatE, *HORVU6Hr1G065390*) and PF00403 (HMA, *HORVU6Hr1G066220*) proteins that may function in Cd transport in the barley seedlings (Table 3 and S3 Table). Taken together, 16 candidate genes related to Cd tolerance at the seedling stage were identified, through the strategy of combining QTL mapping and genome-wide transcriptome analysis.

## Discussion

### Cd-tolerance related QTLs in different populations and cellular tolerance mechanisms

There are few reports of QTLs for Cd tolerance in barley. Recently, a single QTL designated as *qshCd7h*, was identified on chromosome 7H of barley that was linked to shoot Cd concentration. A novel plasma membrane-localized P_1B_-type ATPase gene *HvPAA1* was identified from *qshCd7h* and played a vital role in Cd detoxification [38]. The possible explanation for detecting different QTLs in our study and in the Wang et al. [38] is that the genetic basis of the evaluated trait was different. In our study, the DH lines did not differ in shoot Cd concentration (S2 Fig), whereas in the abovementioned study, a significant phenotypic difference in the DH lines for shoot Cd concentration was reported [38]. In agreement with our results, it has been shown that boron (B) tolerance indicators such as chlorosis and necrosis were not correlated with B uptake and translocation into rice shoots, suggesting that shoot-based tolerance mechanisms played an important role in B toxicity [39]. A possible tolerance mechanism was demonstrated for tolerant cultivars of barley and wheat, in which efflux transporters pump B from the intracellular phase, into the less sensitive apoplast of the leaves [40]. Therefore, we can assume such mechanism for Cd efflux through transporters in the leaves of tolerant genotypes in our study. Moreover, we could not detect any P_1B_-type ATPase related to Cd transport in the detected QTL regions, further supporting the role of Cd detoxification through ABC transporters identified in our study. Another differently designed study using barley genotypes grown on Cd-contaminated hydroponic media, reported 13 QTLs for shoot Cd concentration [41], and among them, a QTL on chromosome 6H (marker *6854–309*) was located between *GBS0822* and *GBM1389* markers on the major QTL in our study. It is reported that the uptake of Cd is in competition with Mn^2+^ through the same transmembrane carrier [42]. Comparison of the major QTL for Cd tolerance in our study with those reported for Mn^2+^ tolerance in barley DH population, revealed that the QTL *QSur*.*yf*.*6H* for plant survival trait mapped near *bPb-3230* marker [43], which was located between *ABG474* and *GBR0029* markers in our study. The results indicated that the existence of similar QTLs might be useful for barley breeding using marker assisted selection and development of tolerant varieties to both Cd and excess Mn^2+^ stresses.

### Candidate genes for Cd tolerance related to the QTL

Integration of QTL mapping and transcriptome profiling has been used to identify candidate genes underlying abiotic stress tolerance in crops [44, 45]. However, such studies have not been reported in barley and could be quite costly when applied to large number of samples. In our study, we tried to reduce the number of samples for RNA-Seq profiling using the two parents of the OWB mapping population with extreme phenotypes for Cd tolerance. Through the platform combining QTL mapping and RNA-Seq analysis, 16 candidate genes were co-localized with QTLs for Cd tolerance. The upregulated DEGs in Rec, including *peroxidase*, *lipoxygenase 2*.*3* (harboring nine missense variants) and *glycine rich RNA-binding protein Grp2A*, located in the major QTL region. Class III peroxidases catalyze the reduction of H_2_O_2_ by taking electrons from various donor compounds such as lignin precursors and secondary metabolites [46]. In the *A*. *thaliana* leaves, significant upregulation of the *lipoxygenase 2* (*LOX2*; *AT3G45140*) under Cd and copper (Cu) stresses was reported [47]. Loxs play critical roles in production of oxylipin signaling molecules, which induce expression of genes involved in biosynthesis of secondary metabolites [35]. The *ATGRP8* (*AT4G39260*) is reported to be rapidly upregulated by peroxide-induced oxidative stress and it is suggested to have a regulatory role in gene expression mechanism [48] (Table 3 and S11 Table). These results are consistent with our findings in KEGG pathway enrichment analysis among upregulated DEGs in Rec.Cd vs Dom.Cd comparison (S9 Table), suggesting the participation of oxidoreductase encoding genes in scavenging ROS and maintaining redox homeostasis [49], and thus protecting Rec genotype from Cd damage.

Among the genes containing variants, we are most interested in the *HORVU6Hr1G019080* gene, an ABC transporter with PDR_assoc domain, located in the major QTL. In *A*. *thaliana*, its homologous gene *AT1G15520* encoding AtPDR12, has been shown to function as a pump to exclude Pb(II) and/or Pb(II)-containing toxic compounds from the cytoplasm and contributes to Pb resistance [50]. Notably, the gene *HORVU6Hr1G019080* showed 56 missense variants in Dom and six frameshift variants in Rec genotype (Table 3 and S11 Table), which may correspond to its different function in Cd exclusion between Rec and Dom. The gene *AT1G63440* is a Cu-transporting P-type ATPase HMA5 and is suggested to be involved in Cu compartmentalization and detoxification [51].

Glutathione peroxidases (GPXs) protect cells against oxidative damage generated by ROS and *AT4G11600* gene encoding AtGPX6 showed strongest responses under most abiotic stresses in the *A*. *thaliana* [52]. We identified genes involved in stress signal transduction harboring SNPs and their corresponding homologs in the *A*. *thaliana* such as *AT3G25250* (*OXI1*) and *AT1G63700* (*MAPKKK4*), indicating the presence of stress signaling genes in the major QTL region [53, 54] (Table 3 and S11 Table).

SNPs and InDels are the basis of most differences between alleles and the major determinant of phenotypic differences [55, 56]. Previous studies demonstrated that a single amino acid mutation in the OsHMA3 led to the hyper-accumulation of Cd in the grain of rice [10, 57]. Thus, we can hypothesize that the identified SNPs and InDels in the genes located on the QTL regions, may affect their expression and function between Rec and Dom under Cd stress.

Among the genes in the function category, AtMTP1 encoded by *AT2G46800* gene, is localized in the vacuolar membrane and proposed to play a role in sequestration of excess Zn into vacuoles [58]. A previous study demonstrated that the *AT3G20870* gene encoding a zinc transporter ZTP29, is involved in response to salt stress through the regulation of zinc levels [59]. In plants, significant interaction between Cd and Zn accumulation has been reported [60], therefore information about related transporters may allow the manipulation of plant mineral homeostasis and heavy metal accumulation. Furthermore, the *HORVU6Hr1G036800* (PDR_assoc) gene, similar to the *HORVU6Hr1G019080* gene, is homologous to the *AT1G15520*, which contributes to Pb resistance [50]. The abovementioned transporters and most other genes in the QTL region were not among the DEGs detected by RNA-Seq analysis, potentially due to differences in the time course of Cd treatment between the RNA-Seq and QTL samples. Consequently, further study of ABC transporter G family member 36 will be valuable for determining Cd-stress mechanisms and in Cd-resistance breeding.

## Conclusion

In summary, the integration of QTL mapping and RNA-Seq analysis assisted us to narrow down the region within the identified QTLs, where the likely underlying candidate genes can be detected. We found that metal ion transporters, antioxidant enzymes and stress signaling proteins showed various kinds of SNPs and InDels between the parents in the QTL regions, which may play crucial roles in Cd stress response. The detected ABC transporters in the QTL region serve as future candidate genes for further gene cloning and breeding Cd-tolerant cultivars in barley.

## Supporting information

S1 Table The order of recombinant markers in each chromosome. (XLSX)

S2 Table Designed primers for selected genes for qRT-PCR analysis. (XLSX)

S3 Table Complete list of identified genes in QTL regions of chromosomes 2H and 6H. (XLSX)

S4 Table Mapping of RNA-Seq reads obtained from shoot samples into the reference *Hordeum_vulgare*. Hv_IBSC_PGSB_v2.dna.toplevel.fa genome sequence.
(XLSX)

S5 Table List of DEGs between treatments (Rec.Cd vs Rec.CK). (XLSX)

S6 Table List of DEGs between treatments (Dom.Cd vs Dom.CK). (XLSX)

S7 Table List of DEGs between genotypes (Rec.Cd vs Dom.Cd). (XLSX)

S8 Table List of DEGs between genotypes (Rec.CK vs Dom.CK). (XLSX)

S9 Table Significantly enriched KEGG pathways for up- and downregulated DEGs in pairwise comparisons. (XLSX)

S10 Table Complete list of identified variants between Rec and Dom. (XLSX)

S11 Table Complete list of DEGs and genes containing variants in QTL regions of chromosomes 2H and 6H. (XLSX)

S1 Fig Phenotypic differences between the OWB parents. (A) The spike phenotype of the OWB lines. (B) Symptoms of Cd stress in Rec and Dom seedlings after nine days growing under 5 mM CdCl_2_-containing Hoagland nutrient solution. The red arrows indicate the Cd-stress symptoms in the tolerant (without the chlorosis and necrosis) and susceptible (with the chlorosis and necrosis) genotypes.
(TIF)

S2 Fig Scatter plot and Pearson’s correlation coefficient between different traits and replicates in the OWB mapping population. The lower panel below the diagonal shows the scatter plots for Cd concentration, chlorosis and necrosis among the OWB population. The upper panel above the diagonal shows Pearson’s correlation coefficient (R) values for the mentioned traits (Cd: Cd concentration).
(TIF)

S3 Fig QTL scanning curves for the chlorosis and necrosis in the OWB population on all chromosomes. The vertical axis represents the distribution of markers. The horizontal axis represents the LOD score. The lower panel represents an additive effect. Ch: Chromosome.
(TIF)

S4 Fig Volcano plots and venn diagram of DEGs in four comparisons. DEGs (A) in Rec genotype, between Cd stress and CK treatment (Rec.Cd vs Rec.CK), (B) in Dom genotype, between Cd stress and CK treatment (Dom.Cd vs Dom.CK), (C) between the two genotypes and under Cd treatment (Rec.Cd vs Dom.Cd), and (D) between the two genotypes and under CK treatment (Rec.CK vs Dom.CK) displayed by volcano plots. The x-axis shows the fold change difference in the expression of genes in four comparisons, and the y-axis indicates the adjusted *p*-values for the differences in expression. Genes without significant differences are indicated by black dots. The upregulated genes are represented by red dots, and the downregulated genes are represented by blue dots. (E) Venn diagram analysis represents the overlapping DEGs number in four comparisons. The numbers in each circle (Rec.Cd vs Rec.CK, Dom.Cd vs Dom.CK, Rec.Cd vs Dom.Cd, Rec.CK vs Dom.CK) illustrate the total number of different genes in each comparison, and the number in the overlapping areas is the number of shared genes between two comparisons. CK: Control.
(TIF)

S5 Fig Validation of expression profile for six randomly selected DEGs by qRT-PCR. The expression profile of six randomly selected DEGs detected by (A) qRT-PCR and (B) RNA-Seq techniques in Rec.Cd vs Dom.Cd, and Rec.CK vs Dom.CK comparisons. The relative expression levels of the selected genes were compared with Dom genotype and normalized using an internal control (*Actin*) and calculated based on the 2^−ΔΔCt^ method. For the qRT-PCR data, the mean ± standard error of three technical replicates are represented. Asterisks indicate levels of significance of differential expression tested by the Student’s t-test (* p ≤ 0.05, ** p ≤ 0.01). Nd: No data.
(TIF)
