# Supplementary figures and images for: Combined QTL mapping and RNA-Seq profiling reveals candidate genes associated with cadmium tolerance in barley
Source: PLoS One. 2020 Apr 16;15(4):e0230820. doi: 10.1371/journal.pone.0230820 (PMC7182363; doi:10.1371/journal.pone.0230820)

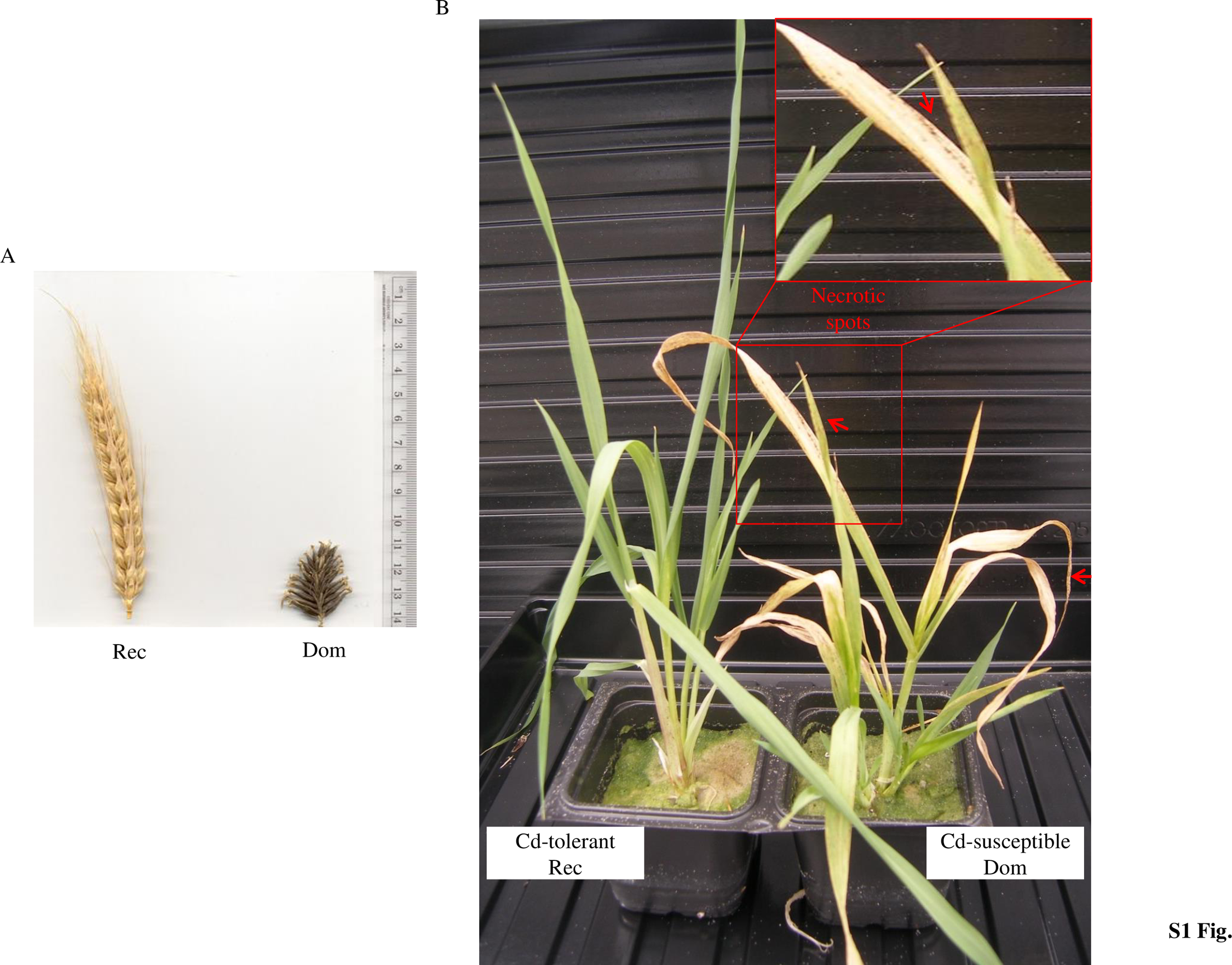

Supplement: S1 Fig — (A) The spike phenotype of the OWB lines. (B) Symptoms of Cd stress in Rec and Dom seedlings after nine days growing under 5 mM CdCl2-containing Hoagland nutrient solution. The red arrows indicate the Cd-stress symptoms in the tolerant (without the chlorosis and necrosis) and susceptible (with the chlorosis and necrosis) genotypes. (TIF) [file pone.0230820.s012.tif]

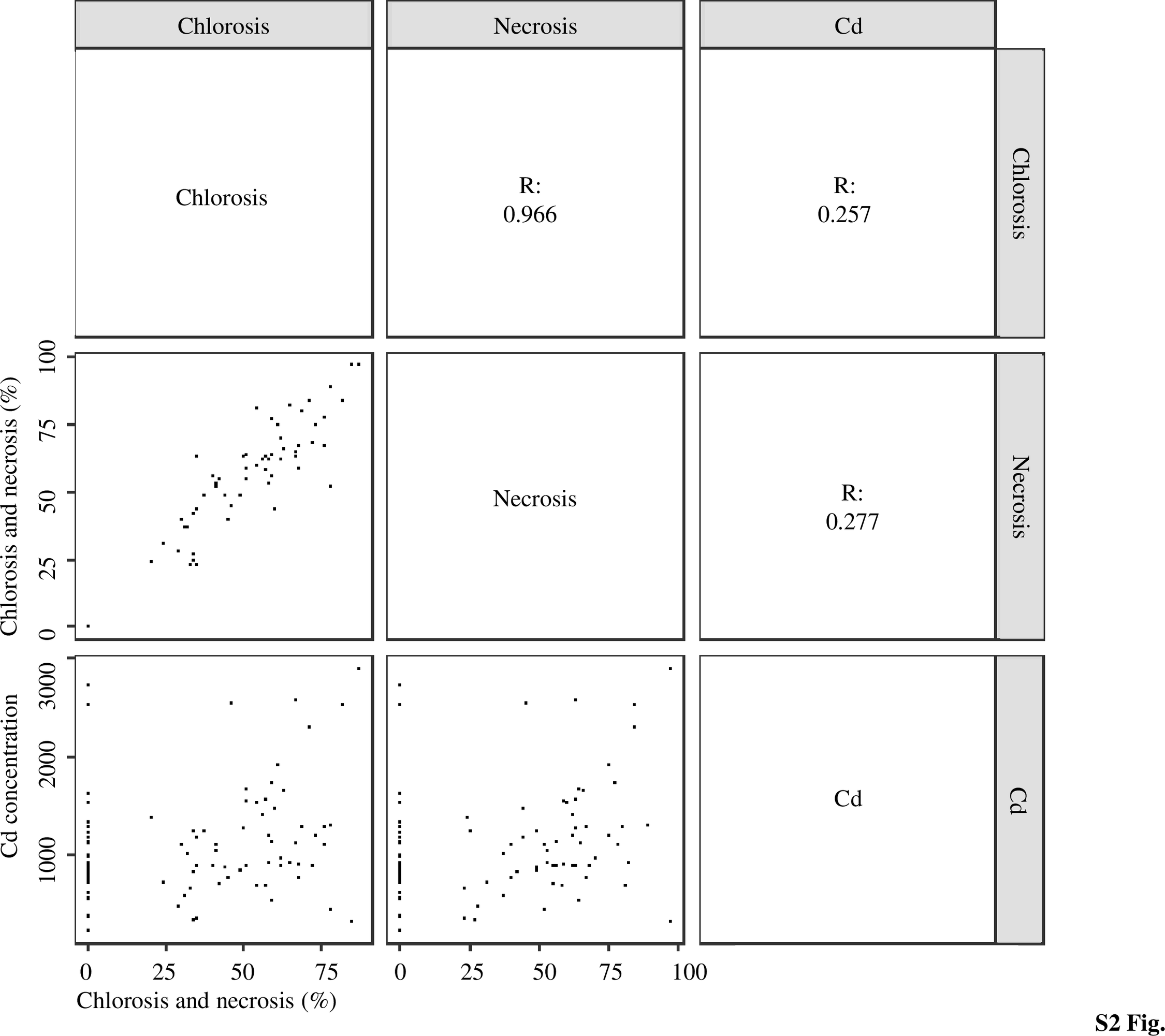

Supplement: S2 Fig — The lower panel below the diagonal shows the scatter plots for Cd concentration, chlorosis and necrosis among the OWB population. The upper panel above the diagonal shows Pearson’s correlation coefficient (R) values for the mentioned traits (Cd: Cd concentration). (TIF) [file pone.0230820.s013.tif]

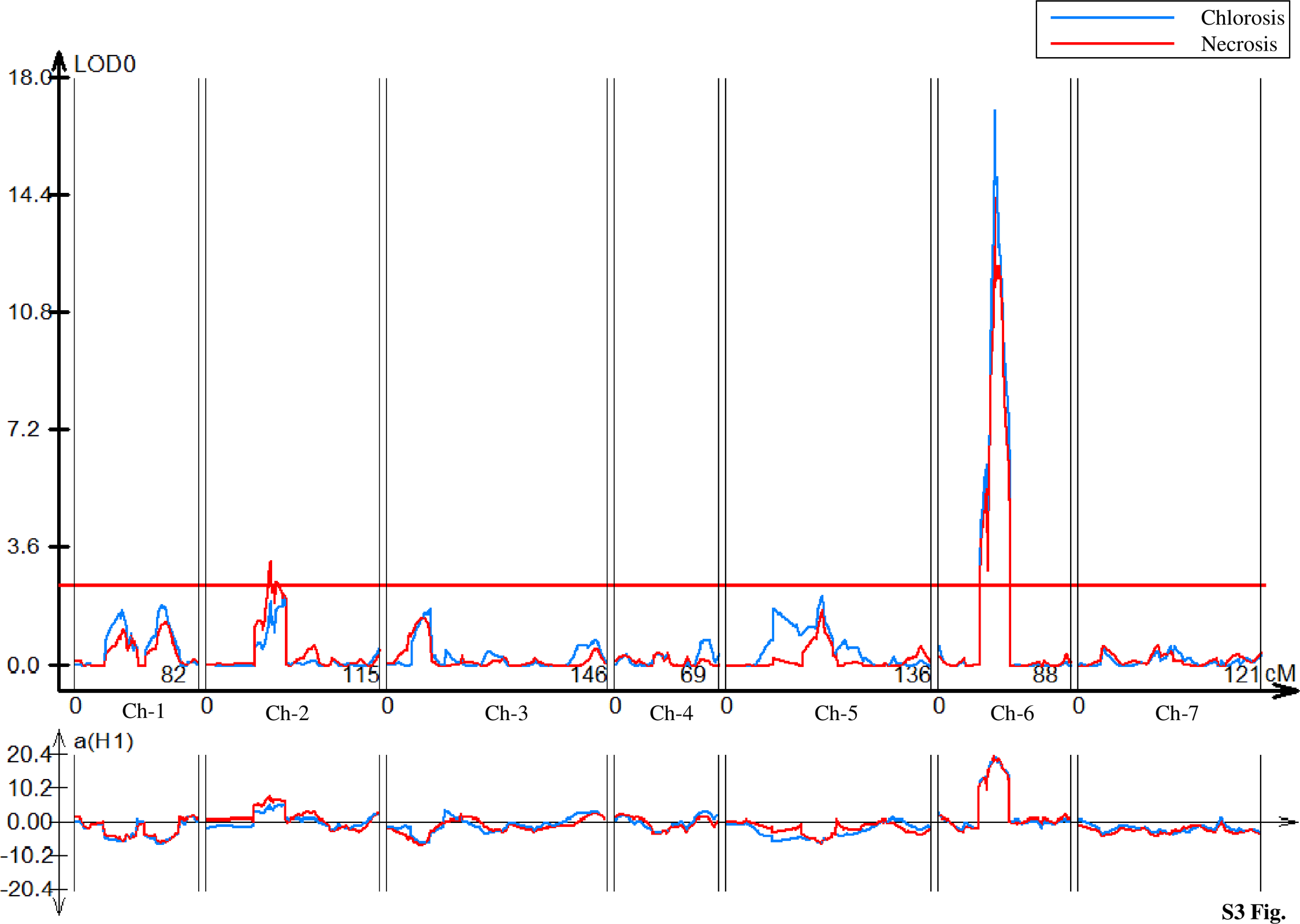

Supplement: S3 Fig — The vertical axis represents the distribution of markers. The horizontal axis represents the LOD score. The lower panel represents an additive effect. Ch: Chromosome. (TIF) [file pone.0230820.s014.tif]

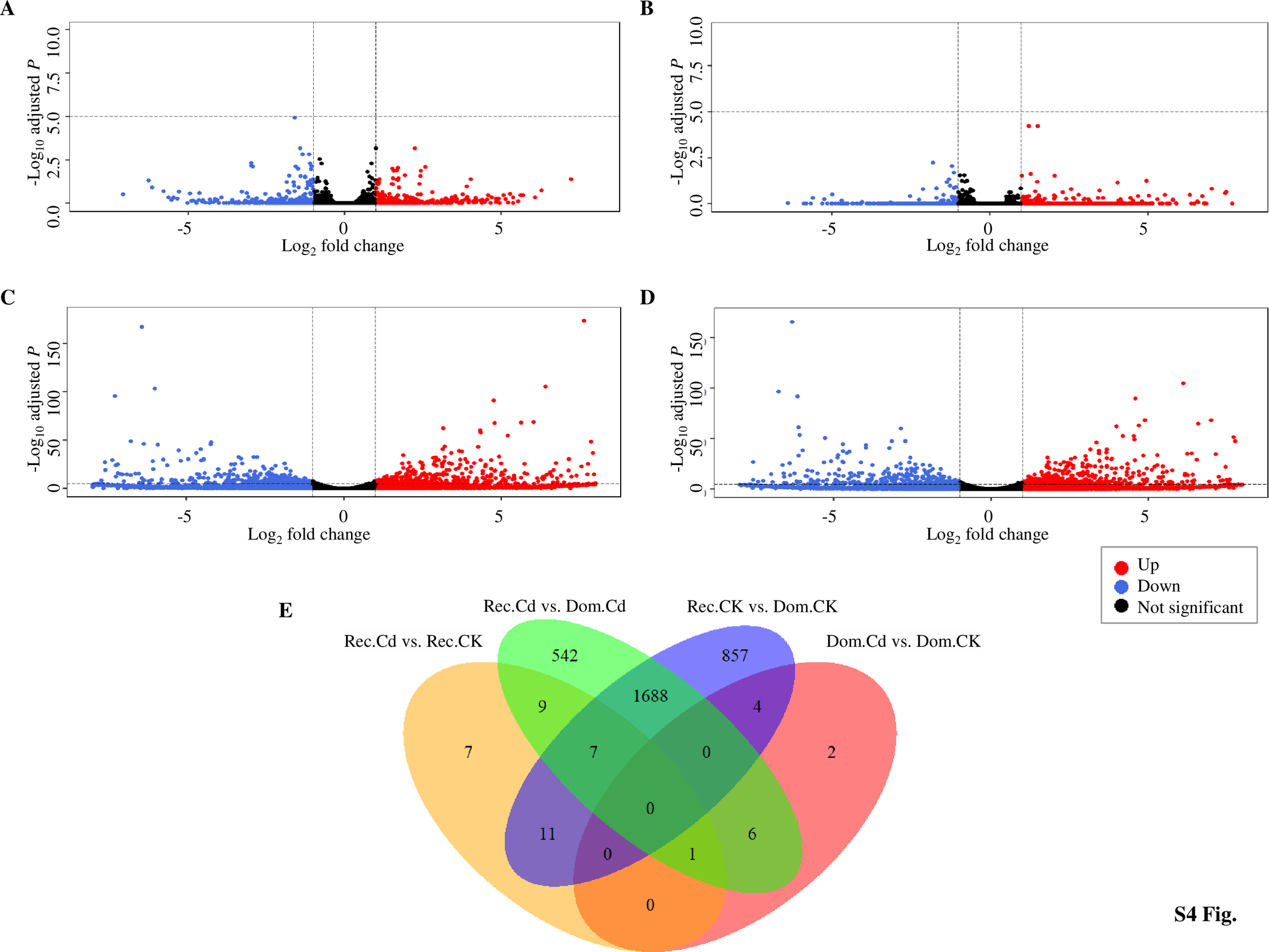

Supplement: S4 Fig — DEGs (A) in Rec genotype, between Cd stress and CK treatment (Rec.Cd vs Rec.CK), (B) in Dom genotype, between Cd stress and CK treatment (Dom.Cd vs Dom.CK), (C) between the two genotypes and under Cd treatment (Rec.Cd vs Dom.Cd), and (D) between the two genotypes and under CK treatment (Rec.CK vs Dom.CK) displayed by volcano plots. The x-axis shows the fold change difference in the expression of genes in four comparisons, and the y-axis indicates the adjusted p-values for the differences in expression. Genes without significant differences are indicated by black dots. The upregulated genes are represented by red dots, and the downregulated genes are represented by blue dots. (E) Venn diagram analysis represents the overlapping DEGs number in four comparisons. The numbers in each circle (Rec.Cd vs Rec.CK, Dom.Cd vs Dom.CK, Rec.Cd vs Dom.Cd, Rec.CK vs Dom.CK) illustrate the total number of different genes in each comparison, and the number in the overlapping areas is the number of shared genes between two comparisons. CK: Control. (TIF) [file pone.0230820.s015.tif]

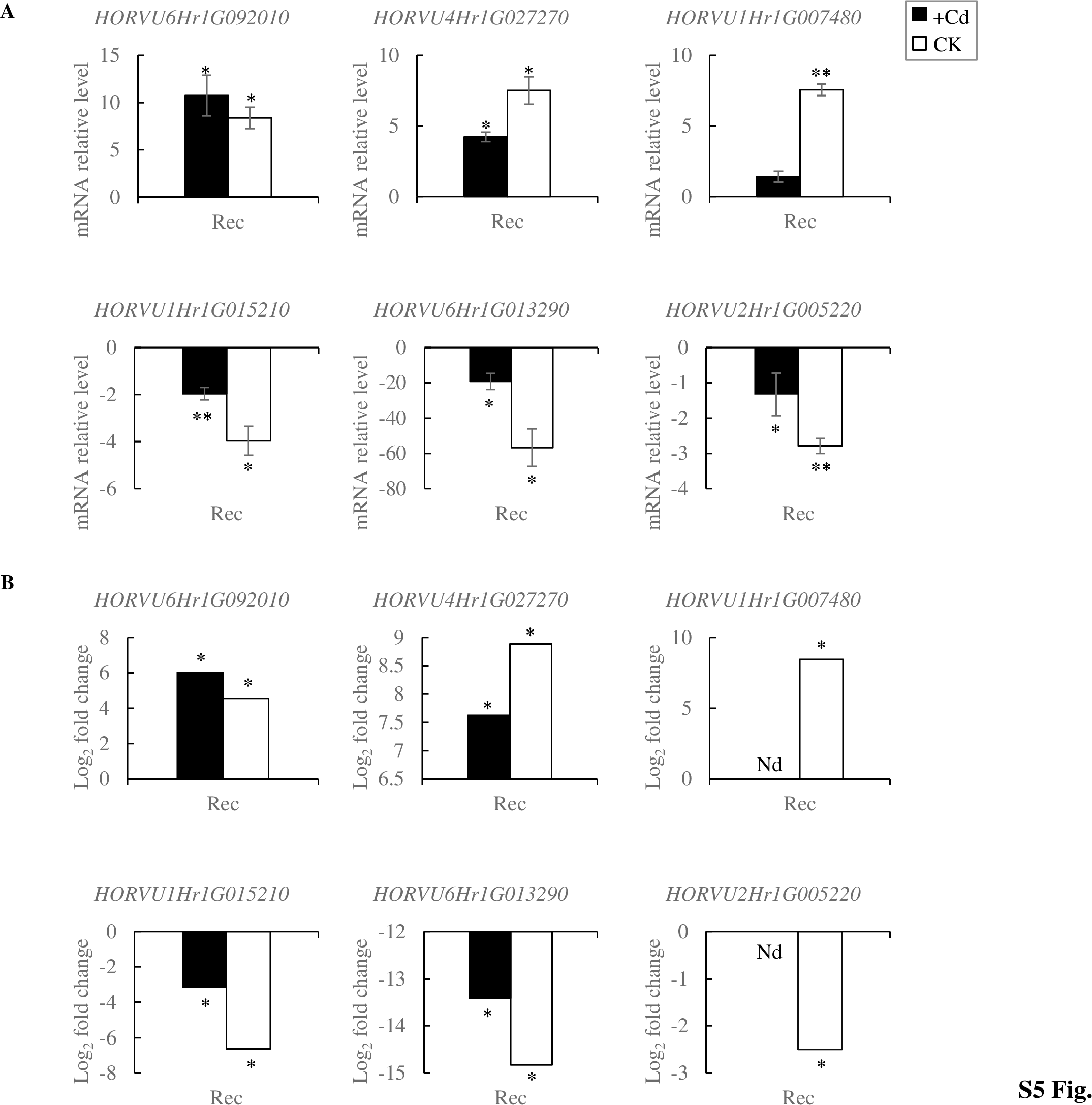

Supplement: S5 Fig — The expression profile of six randomly selected DEGs detected by (A) qRT-PCR and (B) RNA-Seq techniques in Rec.Cd vs Dom.Cd, and Rec.CK vs Dom.CK comparisons. The relative expression levels of the selected genes were compared with Dom genotype and normalized using an internal control (Actin) and calculated based on the 2−ΔΔCt method. For the qRT-PCR data, the mean ± standard error of three technical replicates are represented. Asterisks indicate levels of significance of differential expression tested by the Student’s t-test (* p ≤ 0.05, ** p ≤ 0.01). Nd: No data. (TIF) [file pone.0230820.s016.tif]
